# Certolizumab pegol in a heterogeneous population of patients with moderate-to-severe rheumatoid arthritis

**DOI:** 10.4155/fsoa-2017-0149

**Published:** 2018-02-15

**Authors:** Enrique R Soriano, Analia Dellepiane, Gabriela Salvatierra, Cristian Alejandro Benítez, Rodrigo Garcia Salinas, Carlos Baruzzo

**Affiliations:** 1 Rheumatology Unit, Hospital Italiano de Buenos Aires & Instituto Universitario Hospital Italiano de Buenos Aires, Buenos Aires, Argentina; 2 CEMEC Casilda, Santa Fe, Argentina; 3 Quorum Salud, Buenos Aires, Argentina; 4 Hospital Argerich, Buenos Aires, Argentina; 5 Hospital Italiano de La Plata, Buenos Aires, Argentina; 6 Sociedad Argentina de Reumatología, Buenos Aires, Argentina

**Keywords:** anti-TNFα agent, certolizumab, rheumatoid arthritis

## Abstract

**Aim:**

To determine the efficacy and safety of certolizumab pegol for the treatment of rheumatoid arthritis in a real-world setting.

**Materials & methods:**

Patients with moderate-to-severe rheumatoid arthritis who initiated therapy with certolizumab were followed for 12 weeks. Response was assessed with Disease Activity Score of 28 joints, European Ligue Against Rheumatism criteria and Simplified Disease Activity Index. Predictors of response were analyzed with binary logistic regression models.

**Results:**

Statistically significant decreases in tender and swollen joint counts, laboratory parameters and use of corticosteroids and disease-modifying antirheumatic drugs were found. Disease activity also significantly diminished. Higher Disease Activity Score of 28 joints at baseline was the main predictor of response. No severe adverse events were reported.

**Conclusion:**

Certolizumab was effective and well tolerated, particularly in the subpopulation with higher inflammatory burden at baseline.

## Lay abstract

Rheumatoid arthritis (RA) is a chronic inflammatory disease that destroys cartilage and bone of joints. It is associated with progressive disability and reduced quality of life. However, new biological therapies have improved RA prognosis. Certolizumab pegol, a biological agent licensed for RA treatment, was administered to patients with moderate or severe RA. After 12 weeks, joint pain and inflammation significantly diminished and analytical parameters improved. Certolizumab was particularly effective in patients with higher inflammation at baseline.

Rheumatoid arthritis (RA) is an autoimmune inflammatory disease of unknown etiology [1] characterized by persistent synovitis with cartilage and bone destruction, autoantibodies and systemic features [1,2]. It is associated with progressive disability [1], reduced quality of life and even increased mortality [3]. However, its prognosis and management have changed over the last two decades [4] because of better knowledge of RA pathogenesis and consequent development of new therapies [1].

A key inflammatory pathway in RA includes overproduction and overexpression of TNFα, which are related to synovial inflammation and joint destruction. Anti-TNFα agents were the first licensed biological drugs for RA treatment [2].

Certolizumab pegol (certolizumab) is a PEGylated, humanized, antigen-binding fragment of an anti-TNFα monoclonal antibody [5,6]. Its lack of a crystallized fragment region minimizes potential crystallized fragment-mediated effects while the addition of a PEG moiety improves certolizumab pharmacokinetics and bioavailability [5]. Moreover, its pharmacodynamic properties are different from other anti-TNFα agents [6]. Certolizumab has been extensively studied in randomized clinical trials in RA patients who did not respond to disease-modifying antirheumatic drugs (DMARDs) [7–11], were naive to these agents [12–14] or did not respond to other anti-TNFα agents [15,16]. In addition, certolizumab has been effective and well tolerated in long-term studies [11,17–18] up to almost 6 years of therapy [19]. It is indicated for the treatment of moderate-to-severe RA in adult patients with insufficient response to DMARDs as well as in case of intolerance or contraindication of DMARDs. It is also indicated in patients with severe RA without prior DMARD therapy. Certolizumab can be administered in combination with methotrexate or in monotherapy [20].

The objective of our study was to assess the effectiveness and safety of certolizumab in adult patients with moderate-to-severe RA.

## Materials & methods

A nationwide registry addressing the use of certolizumab in Argentina, the SUPERAR registry, was launched in 2016. It was intended to record and monitor patients over the age of 18 years who were diagnosed with RA according to the 1987 American College of Rheumatology criteria [21] and who initiated certolizumab on a standard clinical care basis. The registry encompasses 35 hospital- and community-based rheumatology units throughout Argentina (Buenos Aires, Santa Fe, Santiago del Estero, Chaco, Entre Ríos, Neuquén and Jujuy). It has been approved by the relevant local ethics committees and patients are enrolled after giving their written informed consent. Certolizumab is used as per the 2013 Clinical Practice Guidelines of the Argentinian Society of Rheumatology for the use of biological therapies in RA [4], which recommended the use of an anti-TNFα in patients with active RA who have failed to respond to two or more DMARDs or to methotrexate monotherapy.

An observational longitudinal prospective study was conducted using the SUPERAR registry. Data were collected at baseline and at the 3-month visit in order to assess effectiveness and safety of certolizumab pegol in RA patients under real-world conditions.

Recorded data at baseline included age, gender, weight, professional activity, working status, smoking habit, diagnosis date, number of prior DMARDs (also known as synthetic DMARDs) and number of prior biological agents (also known as biological DMARDs). Corticosteroid type and dose, types of DMARDs and NSAIDs, tender and swollen joint counts and laboratory parameters were recorded at baseline and after 12 weeks of certolizumab therapy. Start and end dates of certolizumab therapy were registered.

Response to certolizumab was defined in three ways: Disease Activity Score of 28 joints (DAS28) reduction >1.2 points [22], a good/moderate response according to the European Ligue Against Rheumatism (EULAR) criteria [23] and a Simplified Disease Activity Index (SDAI) [24] reduction >16 points at the end of the study [23]. Good EULAR response was defined as a DAS28 score ≤3.2 and reduction in DAS28 >1.2; moderate EULAR response was defined as DAS28 score >3.2 and reduction in DAS28 score >1.2 [23].

Effectiveness population was composed by those patients who initiated certolizumab therapy and had at least one available effectiveness measure. Safety population was composed by all patients who initiated certolizumab therapy.

### Statistical analysis

Descriptive analysis of variables included frequency and percentage for qualitative variables, and mean, SD, median, minimum and maximum for quantitative variables. Variations along the observation period were analyzed with McNemar's test for dichotomic variables and with Wilcoxon's test for quantitative variables. Responses as per baseline variables were analyzed with Student's or Mann–Whitney's test and χ^2^ or Fisher's exact test based on the nature of the variables.

We analyzed the possible relationships between basal variables and patients’ response. Then, we preselected those variables with p < 0.10 and included them in binary logistic regression models to determine basal factors that might forecast the response.

Statistical significance was set at p < 0.05. All analyses were performed using SPSS version 19.0 (SPSS, Inc., IL, USA).

## Results

Table 1 shows the sociodemographic and basal clinical data, as well as prior therapies, of the 247 patients included in the study. They were mostly women, middle age, with comorbidities and a mean RA duration of almost 10 years. More than 90% of patients had received at least one prior DMARD, mainly methotrexate and leflunomide. More than a quarter of patients had been previously treated with at least one prior biological agent, mainly etanercept.

**Table 1.** **Socio-demographic data, basal clinical data and prior therapies.**

**Variable**	**N (%) or mean ± SD**
**Socio-demographic data**	

Gender	

– Male – Female	54 (21.9) 193 (78.1)

Age (years)	55.9 ± 12.5

Weight (kg)	71.3 ± 12.0

**Professional activity**	

– Housewife – White-collar worker – Laborer (manual labor) – Civil servant or employee (nonmanager) – Civil servant or employee (manager) – Student	52 (49.1) 16 (15.1) 16 (15.1) 15 (14.2) 4 (3.8) 3 (2.8)

**Working status**	

– Normal working activity – Temporary work disability – Retirement – Permanent/absolute work disability	33 (64.7) 9 (17.6) 8 (15.7) 1 (2.0)

**Smoking**	

– Never – Current smoker – Ex-smoker	174 (70.7) 43 (17.5) 29 (11.8)

**Clinical data**	

RA duration (years)	9.8 ± 8.1; range: 0–44

Seropositive RA	

– Yes – No	216 (88.9) 27 (11.1)

Erosive RA	

– Yes – No	185 (74.9) 62 (25.1)

Comorbidities	

– Yes – No	156 (79.6) 40 (20.4)

**Prior therapies**	

Number of prior DMARDs	

– 0 – 1 – 2 – 3 – 4	24 (9.8) 88 (35.9) 90 (36.7) 36 (14.7) 7 (2.9)

Prior DMARDs	

– Methotrexate – Leflunomide – Antimalarial drug – Sulfasalazine – Other	155 (63.3) 143 (58.4) 71 (29.0) 32 (13.1) 3 (1.2)

Number of prior biological agents	

– 0 – 1 – 2 – 3 – 4 – 5	161 (67.1) 60 (25.0) 15 (6.3) 1 (0.4) 2 (0.8) 1 (0.4)

Prior biological agents	

– Etanercept – Adalimumab – Golimumab – Abatacept – Tocilizumab – Infliximab – Rituximab	36 (15.0) 33 (13.8) 10 (4.2) 9 (3.8) 9 (3.8) 7 (2.9) 2 (0.8)

DMARD: Disease-modifying antirheumatic drug; RA: Rheumatoid arthritis.

All patients received therapy for 12 weeks. Most patients received certolizumab pegol according to the product label insert: a dose of 400 mg at weeks 0, 2 and 4, followed by 200 mg every 2 weeks. Only 30 patients initiated therapy with 200 mg every 2 weeks without induction phase. Moreover, most patients (96%) received certolizumab combined with corticosteroids, DMARDs and/or NSAIDs. At baseline, 87% of patients received DMARDs (mainly methotrexate), and more than 45% of patients received NSAIDs (mainly diclofenac and naproxen; Table 2).

**Table 2.** **Current therapies.**

**Therapy**	**N (%)**
**Certolizumab loading dose**	

– Yes – No	207 (87.3) 30 (12.7)

**Concomitant drugs**	

Corticosteroids + DMARDs + NSAIDs	79 (32.0)

DMARDs + corticosteroids	74 (30.0)

DMARDs	37 (15.0)

DMARDs + NSAIDs	25 (10.1)

None	10 (4.0)

Corticosteroids	9 (3.6)

Corticosteroids + NSAIDs	9 (3.6)

NSAIDs	4 (1.6)

**Concomitant DMARDs**	

– Methotrexate – Leflunomide – Antimalarial drug – Sulfasalazine	187 (75.7) 34 (13.8) 8 (3.2) 4 (1.6)

**Concomitant NSAIDs**	

– Diclofenac – Naproxen – Ibuprofen – Etoricoxib – Indomethacin – Celecoxib – Other	62 (25.1) 38 (15.4) 26 (10.5) 4 (1.6) 2 (0.8) 1 (0.4) 2 (0.8)

DMARD: Disease-modifying antirheumatic drug.

At the 3-month visit, certolizumab was discontinued in seven patients (2.8%) due to lack of efficacy and in three patients (1.2%) due to adverse events (AEs).

Changes in joint disease, laboratory parameters and drug therapies after 12 weeks of certolizumab treatment are summarized in Table 3. Tender and swollen joint counts significantly decreased. Rheumatoid factor (RF), anticyclic citrullinated peptide (anti-CCP), C-reactive protein (CRP) and erythrocyte sedimentation rate (ESR) values also diminished. Moreover, corticosteroid therapy was reduced in both mean dose and number of patients. Use of DMARDs and NSAIDs was also reduced.

**Table 3.** **Changes in joint disease, laboratory parameters and drug therapies.**

	**Basal**	**After 3 months**	**p-value**
**Joint disease (mean ± SD)**			

– Tender joint count	10.4 ± 5.8; 10.0	4.0 ± 3.4	<0.001

– Swollen joint count	7.5 ± 4.6; 6.0	2.4 ± 2.6	<0.001

**Laboratory parameters (mean ± SD)**			

– RF (IU/ml)	211.7 ± 210.9	170.4 ± 217.9	<0.001

– Anti-CCP (IU/ml)	220.9 ± 212.5	163.3 ± 168.9	0.020

– CRP (mg/dl)	21.9 ± 25.1	10.8 ± 16.5	<0.001

– ESR (mm/h)	45.8 ± 23.0	27.6 ± 16.7	<0.001

**Concomitant therapies**			

Corticosteroids (n, %)			

– Yes – No	171 (69.2) 76 (30.8)	104 (42.1) 143 (57.9)	<0.001

Corticosteroid dose (mg/day)	7.4 ± 4.4	3.3 ± 3.4	<0.001

DMARDs (n, %)			

– 0 – 1 – 2	32 (13.09) 197 (79.8) 18 (7.3)	34 (13.8) 201 (81.4) 12 (4.9)	0.033

NSAIDs (n, %)			

– 0 – 1 – 2 – 3	130 (52.6) 102 (41.3) 12 (4.9) 3 (1.2)	128 (51.8) 115 (46.6) 4 (1.6) 0 (0.0)	0.023

Anti-CCP: Anticyclic citrullinated peptide antibody; CRP: C-reactive protein; DMARD: Disease-modifying antirheumatic drug; ESR: Erythrocyte sedimentation rate; RF: Rheumatoid factor; SD: Standard deviation.

Disease activity, as measured by DAS28 and SDAI, significantly diminished compared with baseline. Percentages of patients with high RA activity also decreased (Table 4). In addition, response as per EULAR was good in 40 patients (17.2%) and moderate in 162 patients (69.5%).

**Table 4.** **Changes in disease activity.**

**Index**	**Basal**	**After 3 months**	**p-value**

	**N (%) or mean ± SD**	**N (%) or mean ± SD**	
**DAS28**			

Score (mean ± SD)	5.9 ± 1.0	4.0 ± 1.0	<0.001

RA activity (n, %)			

– Remission – Low – Moderate – High	1 (0.4) 0 (0.0) 41 (17.6) 191 (82.0)	18 (7.7) 27 (11.6) 161 (69.1) 27 (11.6)	<0.001

**SDAI**			

Score (mean ± SD)	50 ± 29.4	22.8 ± 13.0	<0.001

RA activity (n, %)			

– Remission – Low – Moderate – High	0 (0.0) 1 (1.0) 7 (7.1) 91 (91.9)	0 (0.0) 184 (18.2) 44 (44.4) 37 (37.4)	<0.001

DAS28: Disease Activity Score for 28 joint; RA: Rheumatoid arthritis; SD: Standard deviation; SDAI: Simplified Disease Activity Index.

Seven patients (2.8%) had an AE, but only in four patients (1.7%) the AE was considered related to certolizumab (alopecia, moderate rash, moderate herpes zoster and unspecified). No severe AEs were reported.

### Bivariate analysis

After 12 weeks of certolizumab therapy, 174 patients (74.7%; n = 233) were responders as per DAS28, 202 patients (86.7%; n = 233) as per EULAR and 65 patients (65.7%; n = 99) as per SDAI.

Regarding DAS28 response criterion, statistically significant differences were found in the response to certolizumab by RA duration, which was significantly lower in responders (9.1 years vs 11.8 years in nonresponder patients; p = 0.021). Moreover, 70.38% of responders were naive to biological agents versus 49.1% of nonresponders (p = 0.003). Tender joint count and swollen joint count at baseline were higher in responders (p < 0.001 for both). Basal CRP and ESR were significantly higher in responders (p = 0.027 and p = 0.026, respectively). Mean DAS28 score at baseline was higher in responders (6.1 ± 0.9) versus nonresponders (5.2 ± 0.9; p < 0.001). See detailed data in the Supplementary Material.

Moreover, 202 patients (86.7%) were defined as responders as per EULAR response criterion (good or moderate response). Basal tender joint count was higher in responders (p = 0.007), as well as basal swollen joint count (p = 0.004). Responders also had higher RF (p = 0.029) and CRP (p = 0.030) values, as well as DAS28 score (p = 0.001). See detailed data in the Supplementary Material.

Finally, for SDAI response (a decrease >16 points), basal tender joint count was higher in responders (p = 0.005), as well as basal swollen joint count (p < 0.001), CRP (p < 0.001), ESR (p = 0.002) and DAS28 (p < 0.001). See detailed data in the Supplementary Material.

### Binary logistic regression analysis

For the DAS28 model, the best outcome model maintained ‘Number of prior biological agents’ and ‘Baseline DAS28’. The lower the number of prior biological agents, the higher the probability of being responder. The inversion of odds ratio (OR: 1.787; 95% CI: 1.21–1.63) reflected that it was more than 1.5 more likely to be nonresponder by each unit of increase of the number of prior biological agents. Moreover, the higher the basal DAS28 score, the higher the probability of being responder. The OR reflected that it was 3.3 more likely to be responder by each unit of basal DAS28 increase (Table 5).

**Table 5.** **Binary logistic regression analysis.**

	**Coefficient**	**SE**	**OR**	**95% CI**	**p-value**
**DAS28 response**					

– Number of prior biological agents	-0.581	0.198	0.559	(0.38; 0.82)	0.003

– Basal DAS28	1.199	0.224	3.315	(2.14; 5.14)	<0.001

**EULAR response**					

Basal DAS28	1.101	0.269	3.006	(1.73; 5.10)	<0.001

DAS28: Disease Activity Score for 28 joint; EULAR: European Ligue Against Rheumatism; OR: Odds ratio; SE: Standard error.

Regarding SDAI response, the only variable that could be selected for binary regression model was the basal DAS28 score. Therefore, we could not obtain a multivariate model to predict response to certolizumab as per the SDAI response criterion.

## Discussion

We have presented the results of a registry-based study of certolizumab in RA patients. As opposite to clinical studies, registries generally include heterogeneous patient populations, and therefore, registry-based studies have more generalizable results [25]. In our real-world clinical practice study, certolizumab pegol for 12 weeks was effective and well tolerated in a heterogeneous group of patients with moderate-to-severe RA, including almost 10% of DMARD-naive patients and more than 25% previously treated with other biological agent. Tender and swollen joint counts diminished, acute-phase reactants and, interestingly, autoantibodies titers (RF and anti-CCP) improved and use of corticosteroids, DMARDs and NSAIDs decreased. RA activity, measured with both DAS28 and SDAI scores, diminished. Percentage of patients with high RA activity was also reduced. Moreover, response as per EULAR was good or moderate in more than 80% of patients. In addition, the incidence of AEs was low and no severe AE was reported.

Other real-world studies of certolizumab in RA patients have also found significant improvements. Two of them were registry-based studies in Sweden [26] and Italy [27] and the third was a Spanish observational multicenter study [28]. Study designs were different and EULAR response was evaluated at 6 months [26,27] and/or 1 year [27,28]. Although certolizumab was effective and safe in all these studies, percentages of good response were different: 38 [26] and 65% [27] of patients at 6 months and 66 [27] and 46.4% [28] of patients at 12 months. These differences could reflect the heterogeneity of populations.

In our study, the number of prior biological therapies was a predictor of response as per DAS28: response was less likely as the number of prior biological therapies increased. The other real-world studies found similar results. Patients naive to anti-TNF agents had better results at 6 months [26] and had adjusted OR of good EULAR response at 3 months sixfold higher than in those with at least one prior biological agent [27]. In the Spanish study [28], lower number of prior biological therapies was a predictor of response. Failure of a first biological agent can be primary or secondary, or can be due to AEs [29]. Certolizumab has been shown effective as second anti-TNF agent, as in a double blind, randomized trial in patients who were secondary nonresponders to other anti-TNF agents [15]. Moreover, in a randomized clinical trial, patients who did not respond to adalimumab were switched to certolizumab without a washout period; 58% of patients achieved low disease activity (DAS28 ≤3.2) or DAS28 reduction of at least 1.2 points from baseline [30]. However, management of patients with an inadequate response to a first biological agent is still not clear and more studies are needed [29]. We did not analyze response by prior biological agent or by type of nonresponders. This was beyond the scope of our study.

Another predictor of response was DAS28 score at baseline. Traditionally, it has been considered that lower disease activity at baseline is a predictor of clinical efficacy of anti-TNF agents in RA [31–34]. However, in our study, patients with higher disease activity (DAS28) at baseline were more responsive to certolizumab, both as per DAS28 and EULAR criteria. A similar result was found in an observational, prospective multicenter study, although basal DAS28 was predictor of response only as per DAS28 [28]. This unexpected result could be related to the different effect of the four DAS28 components on total score, with the greatest effect for ESR [35] and tender joint count [36]. In fact, in our study, responders had higher ESR. Because of these differences, DAS28 could suggest a false high disease activity [37].

One limitation of our study can be its duration. We chose a 3-month period because clinical response to certolizumab is usually achieved within the first 12 weeks of therapy [38]. In addition, nonresponse to certolizumab at 12 weeks predicted treatment failure at 1 year [38,39], whereas a good response to certolizumab within this period has been shown to predict low disease activity at 1 year in clinical trials [38,40–41] and registry-based studies [27].

A long-term follow-up of our patients is underway. We hope to obtain more data regarding predictors of response and to add evidence to the relevance of early response to certolizumab.

We conclude that therapy with certolizumab pegol for 12 weeks significantly decreased clinical (tender and inflammatory joint counts) and biological (RF, anti-CCP, ESR and CRP) activity in our patients with RA, particularly in the subpopulation with higher inflammatory burden at baseline.

## Future perspective

The ultimate goal of RA treatment should be disease remission. Current therapies have improved RA prognosis and biological agents such as anti-TNFs are effective in most RA patients. However, remission rates are still unsatisfactory [42]. Therefore, new RA therapies are in development. These potential new treatments include inhibitors of the granulocyte macrophage-colony stimulating factor pathway, as well as inhibitors of several intracellular kinase pathways: the Janus kinase-signal transducer and activator of transcription, the Bruton's tyrosine kinase and PI3K. Neural stimulation and dendritic cell-based therapeutics are also being investigated [42].

A very interesting field is RA prevention. RA-related systemic autoimmune and inflammatory events occur well before clinical RA. Thus, multiple biomarkers are under study to achieve identification of individuals at risk of RA in general population [43]. Biomarkers can be related to RA phenotypes. For instance, antifibrillar collagen type II antibodies could define a new RA phenotype. Moreover, a combined analysis of antifibrillar collagen type II and anti-CCP antibodies could predict the disease course [44]. There is a trend to personalized RA risk assessment using genetics, biomarkers and lifestyle factors [45]. RA prevention will not be based only in lifestyle changes, because immunomodulators are under study for use in individuals with high risk of RA development [43].

**Summary points**

- Therapy with certolizumab pegol for 12 weeks significantly decreased clinical and biological activity of rheumatoid arthritis.
- Certolizumab pegol was particularly effective in patients with higher Disease Activity Score of 28 joints at baseline.
- Response to certolizumab was less likely if patients had received prior biological agents.
- In our real-world clinical practice study, certolizumab pegol was effective and well tolerated in a heterogeneous group of patients with moderate-to-severe rheumatoid arthritis.
