# Supplementary material for: Certolizumab pegol in a heterogeneous population of patients with moderate-to-severe rheumatoid arthritis
Source: Future Sci OA. 2018 Feb 15;4(4):FSO289. doi: 10.4155/fsoa-2017-0149 (PMC5905631; doi:10.4155/fsoa-2017-0149)
Supplement: Supplementary file 1 [file fsoa-04-289-s1.docx]

| **Table S1. Bivariate analysis: response according to DAS28** | | | |
| --- | --- | --- | --- |
|  | **Responders (n=174)**  **N (%) or mean ± SD** | **Non-responders (n=59)**  **N (%) or mean ± SD** | **p** |
| Gender  Male  Female | 37 (21.3)  137 (78.7) | 13 (22.0)  46 (78.0) | 0.901 |
| Age (y) | 55.2 ± 12.5 | 57.6 ± 11.7 | 0.204 |
| Weight (Kg) | 71.5 ±12.3 | 71.5 ± 12.0 | 0.993 |
| Professional activity  Housewife  Student  White-collar worker  Civil servant or employee (manager)  Civil servant or employee (non manager)  Labourer (manual labour) | 39 (51.3)  2 (2.6)  10 (13.2)  4 (5.3)  10 (13.2)  11 (14.5) | 9 (39.1)  1 (4.3)  5 (21.7)  0 (0.0)  3 (13.0)  5 (21.7) | NA^a^ |
| Working status  Normal working activity  Temporary work disability  Permanent/absolute work disability | 22 (62.9)  7 (20.0)  0 (0.0) | 9 (69.2)  2 (15.4)  1 (7.7) | NA^b^ |
| Smoking  Never  Ex-smoker  Smoker | 127 (73.0)  20 (1.5)  27 (15.5) | 38 (65.5)  8 (13.8)  12 (20.7) | 0.542 |
| RA duration (y) | 9.1 ± 8.1 | 11.8 ± 8.5 | 0.021 |
| Seropositive RA  No  Yes | 21 (12.3)  150 (87.7) | 6 (10.3)  52 (89.7) | 0.693 |
| Erosive RA  No  Yes | 47 (27.0)  127 (73.0) | 13 (22.0)  46 (78.0) | 0.450 |
| Comorbidities  No  Yes | 31 (21.8)  111 (78.2) | 7 (15.6)  38 (84.4) | 0.362 |
| Number of prior DMARDs  0  1  2  3  4 | 22 (12.6)  56 (32.2)  62 (35.6)  27 (15.5)  7 (4.0) | 1 (1.7)  27 (45.8)  23 (39.0)  8 (13.6)  0 (0.0) | 0.036 |
| Prior DMARDs | 1.7 ± 1.0 | 1.6 ± 0.7 | 0.931 |
| Number of prior biological agents  0  1  2  3  4  5 | 121 (70.8)  41 (24.0)  7 (4.1)  0 (0.0)  1 (0.6)  1 (0.6) | 28 (49.1)  19 (33.3)  8 (14.0)  1 (1.8)  1 (1.8)  0 (0.0) | NA^c^ |
| Prior biological agents | 0.4 ± 0.7 | 0.7 ± 0.9 | 0.001 |
| Naïve to biological agents  No  Yes | 50 (29.2)  121 (70.8) | 29 (50.9)  28 (49.1) | 0.003 |
| Corticosteroids  No  Yes | 53 (30.5)  121 (69.5) | 16 (27.1)  43 (72.9) | 0.627 |
| Number of basal DMARDs  0  1  2 | 21 (12.1)  142 (81.6)  11 (6.3) | 5 (8.5)  48 (81.4)  6 (10.2) | 0.495 |
| Number of basal NSAIDs  0  1  2  3 | 89 (51.1)  73 (42.0)  9 (5.2)  3 (1.7) | 32 (54.2)  24 (40.7)  3 (5.1)  0 (0.0) | NA^d^ |
| Induction  No  Yes | 20 (11.7)  151 (88.3) | 8 (14.3)  48 (85.7) | 0.609 |
| Tender joint count | 11.1 ± 5.7 | 7.4 ± 3.9 | <0.001 |
| Swollen joint count | 8.2 ± 4.3 | 4.9 ± 3.2 | <0.001 |
| RF | 202.3 ± 196.0 | 229.6 ± 232.8 | 0.275 |
| Anti-CCP | 204.8 ± 196.5 | 250.8 ± 266.9 | 0.564 |
| CRP | 23.3 ± 24.7 | 18.4 ± 27.8 | 0.027 |
| ESR | 47.5 ± 122.8 | 39.2 ± 20.6 | 0.026 |
| Basal DAS28 | 6.1 ± 0.9 | 5.2 ± 0.9 | <0.001 |

^a^No significance test was carried out because the expected frequency of 58% of cells was less than 5.

^b^No significance test was carried out because the expected frequency of 50% of cells was less than 5.

^c^No significance test was carried out because the expected frequency of 58% of cells was less than 5.

^d^No significance test was carried out because the expected frequency of 38% of cells was less than 5.

RA, rheumatoid arthritis; DMARD, disease-modifying anti-rheumatic drug; NSAID, non-steroid anti-inflammatory drug; RF, rheumatoid factor; Anti-CCP, anti-cyclic citrullinated peptide antibodies; CRP, C-reactive protein; ESR, erythrocyte sedimentation rate; DAS28, Disease activity score; NA, not available.

| **Table S2. Bivariate analysis: response according to EULAR** | | | |
| --- | --- | --- | --- |
|  | **Responders (n=202)**  **N (%) or mean ± SD** | **Non-responders (n=31)**  **N (%) or mean ± SD** | **p** |
| Gender  Male  Female | 40 (19.8)  162 (80.2) | 10 (32.3)  21 (67.7) | 0.116 |
| Age (y) | 55.6 ± 12.6 | 57.3 ± 11.1 | 0.467 |
| Weight (Kg) | 71.1 ± 11.9 | 73.7 ± 13.8 | 0.298 |
| Professional activity  Housewife  Student  White-collar worker  Civil servant or employee (manager)  Civil servant or employee (non manager)  Labourer (manual labour) | 43 (50.6)  3 (3.5)  12 (14.1)  4 (4.7)  11 (12.9)  12 (14.1) | 5 (35.7)  0 (0.0)  3 (21.4)  0 (0.0)  2 (14.3)  4 (28.6) | NA^a^ |
| Working status  Normal working activity  Temporary work disability  Permanent/absolute work disability  Retirement | 26 (66.7)  7 (17.9)  0 (0.0)  6 (15.4) | 5 (55.6)  2 (22.2)  1 (11.1)  1 (11.1) | NA^b^ |
| Smoking  Never  Ex-smoker  Smoker | 144 (71.6)  25 (12.4)  32 (15.9) | 21 (67.7)  3 (9.7)  7 (22.6) | 0.626 |
| RA duration (y) | 9.6 ± 8.2 | 10.9 ± 8.4 | 0.390 |
| Seropositive RA  No  Yes | 26 (13.1)  172 (86.9) | 1 (3.2)  30 (96.8) | 0.140 |
| Erosive RA  No  Yes | 51 (25.2)  151 (74.8) | 9 (29.0)  2 (71.0) | 0.654 |
| Comorbidities  No  Yes | 34 (21.1)  127 (78.9) | 4 (15.4)  22 (84.6) | 0.500 |
| Number of prior DMARDs  0  1  2  3  4 | 22 (10.9)  68 (33.7)  73 (36.1)  32 (15.8)  7 (3.5) | 1 (3.2)  15 (48.4)  12 (38.7)  3 (9.7)  0 (0.0) | NA^c^ |
| Prior DMARDs | 1.7 ± 1.0 | 1.5 ॵ 0.7 | 0.498 |
| Number of prior biological agents  0  1  2  3  4  5 | 133 (67.5)  50 (25.4)  11 (5.6)  1 (0.5)  1 (0.5)  1 (0.5) | 16 (51.6)  10 (32.3)  4 (12.9)  0 (0.0)  1 (3.2)  0 (0.0) | NA^a^ |
| Prior biological agents | 0.4 ± 0.7 | 0.7 ± 0.9 | 0.059 |
| Naïve to biological agents  No  Yes | 64 (32.5)  133 (67.5) | 15 (48.4)  16 (51.6) | 0.084 |
| Corticosteroids  No  Yes | 60 (29.7)  142 (70.3) | 9 (29.0)  22 (71.0) | 0.939 |
| Number of basal DMARDs  0  1  2 | 22 (10.9)  165 (81.7)  15 (7.4) | 4 (12.9)  25 (80.6)  2 (6.5) | NA^a^ |
| Number of basal NSAIDs  0  1  2  3 | 108 (53.5)  82 (40.6)  9 (4.5)  3 (1.5) | 13 (41.9)  15 (48.4)  3 (9.7)  0 (0.0) | NA^d^ |
| Induction  No  Yes | 24 (12.1)  175 (87.9) | 4 (14.3)  24 (85.7) | 0.759 |
| Tender joint count | 10.6 ± 5.5 | 7.7 ± 5.0 | 0.007 |
| Swollen joint count | 7.7 ± 4.2 | 5.2 ± 3.9 | 0.004 |
| RF | 202.0 ± 202.1 | 254.6 ± 228.5 | 0.029 |
| Anti-CCP | 212.9 ± 213.4 | 242.6 ± 235.0 | 0.674 |
| CRP | 23.4 ± 26.8 | 13.4 ± 11.7 | 0.030 |
| ESR | 46.3 ± 22.6 | 39.4 ± 21.6 | 0.157 |
| Basal DAS28 | 6.0 ± 0.9 | 5.2 ± 1.2 | 0.001 |

^a^No significance test was carried out because the expected frequency of 58% of cells was less than 5.

^b^No significance test was carried out because the expected frequency of 50% of cells was less than 5.

^c^No significance test was carried out because the expected frequency of 30% of cells was less than 5.

^d^No significance test was carried out because the expected frequency of 38% of cells was less than 5.

RA, rheumatoid arthritis; DMARD, disease-modifying anti-rheumatic drug; NSAID, non-steroid anti-inflammatory drug; RF, rheumatoid factor; Anti-CCP, anti-cyclic citrullinated peptide antibodies; CRP, C-reactive protein; ESR, erythrocyte sedimentation rate; DAS28, Disease Activity Score

| **Table S3. Bivariate analysis: response according to SDAI** | | | |
| --- | --- | --- | --- |
|  | **Responders**  **(n=65)**  **N (%) or mean ± SD** | **Non-responders (n=34)**  **N (%) or mean ± SD** | **p** |
| Gender  Male  Female | 14 (21.5)  51 (78.5) | 7 (20.6)  27 (79.4) | 0.913 |
| Age (y) | 55.9 ± 12.8 | 56.9 ± 13.3 | 0.717 |
| Weight (Kg) | 72.4 ± 12.5 | 72.3 ± 11.5 | 0.973 |
| Professional activity  Housewife  Student  White-collar worker  Civil servant or employee (manager)  Civil servant or employee (non manager)  Labourer (manual labour) | 16 (51.6)  (0.0)  3 (9.7)  2 (6.5)  5 (16.1)  5 (16.1) | 7 (46.7)  1 (6.7)  3 (20.0)  0 (0.0)  1 (6.7)  3 (20.0) | NA^a^ |
| Working status  Normal working activity  Temporary work disability  Permanent/absolute work disability  Retirement | 12 (80.0)  1 (6.7)  2 (13.3)  12 (80.0) | 4 (57.1)  2 (28.6)  1 (14.3)  4 (57.1) | NA^b^ |
| Smoking  Never  Ex-smoker  Smoker | 48 (73.8)  5 (7.7)  12 (18.5) | 21 (63.6)  6 (18.2)  6 (18.2) | 0.291 |
| RA duration (y) | 9.7 ± 9.2 | 10.0 ± 7.6 | 0.490 |
| Seropositive RA  No  Yes | 4 (6.3)  59 (93.7) | 0 (0.0)  34 (100.0) | 0.294 |
| Erosive RA  No  Yes | 13 (20.0)  52 (80.0) | 8 (23.5)  26 (76.5) | 0.683 |
| Comorbidities  No  Yes | 14 (27.5)  37 (72.5) | 6 (24.0)  19 (76.0) | 0.748 |
| Number of prior DMARDs  0  1  2  3  4 | 6 (9.2)  20 (30.8)  25 (38.5)  13 (20.0)  1 (1.5) | 0 (0.0)  15 (44.1)  15 (44.1)  4 (11.8)  0 (0.0) | NA^c^ |
| Prior DMARDs | 1.7 ± 0.9 | 1.7 ± 0.7 | 0.652 |
| Number of prior biological agents  0  1  2  5 | 45 (72.63)  15 (24.2)  1 (1.6)  1 (1.6) | 24 (72.7)  6 (18.2)  3 (9.1)  0 (0.0) | NA^d^ |
| Prior biological agents | 0.4 ± 0.8 | 0.4 ± 0.7 | 0.892 |
| Naïve to biological agents  No  Yes | 17 (27.4)  45 (72.6) | 9 (27.3)  24 (72.7) | 0.988 |
| Corticosteroids  No  Yes | 12 (18.5)  53 (81.5) | 6 (17.6)  28 (82.4) | 0.921 |
| Number of basal DMARDs  0  1  2 | 7 (10.8)  49 (75.4)  9 (13.8) | 1 (2.9)  30 (88.2)  3 (8.8) | NA^e^ |
| Number of basal NSAIDs  0  1  2  3 | 32 (49.2)  28 (43.1)  3 (4.6)  2 (3.1) | 17 (50.0)  15 (44.1)  2 (5.9)  0 (0.0) | NA^d^ |
| Induction  No  Yes | 4 (6.2)  61 (93.8) | 2 (6.1)  31 (93.9) | 1.000 |
| Tender joint count | 12.6 ± 6.5 | 8.8 ± 4.8 | 0.005 |
| Swollen joint count | 8.3 ± 4.6 | 5.1 ± 3.7 | <0.001 |
| RF | 234.9 ± 235.8 | 200.4 ± 133.5 | 0.634 |
| Anti-CCP | 236.8 ± 232.1 | 195.5 ± 186.4 | 0.703 |
| CRP | 23.4 ± 26.7 | 11.0 ± 10.1 | <0.001 |
| ESR | 49.6 ±22.5 | 35.1 ± 18.8 | 0.002 |
| Basal DAS28 | 6.3 ± 0.9 | 5.3 ± 1.0 | <0.001 |

^a^No significance test was carried out because the expected frequency of 75% of cells was less than 5.

^b^No significance test was carried out because the expected frequency of 67% of cells was less than 5.

^c^No significance test was carried out because the expected frequency of 40% of cells was less than 5.

^d^No significance test was carried out because the expected frequency of 50% of cells was less than 5.

^e^No significance test was carried out because the expected frequency of 33% of cells was less than 5.

RA, rheumatoid arthritis; DMARD, disease-modifying anti-rheumatic drug; NSAID, non-steroid anti-inflammatory drug; RF, rheumatoid factor; Anti-CCP, anti-cyclic citrullinated peptide antibodies; CRP, C-reactive protein; ESR, erythrocyte sedimentation rate; DAS28, Disease Activity Score.
