# Supplementary material for: Certolizumab pegol in a heterogeneous population of patients with moderate-to-severe rheumatoid arthritis
Source: Future Sci OA. 2018 Feb 15;4(4):FSO289. doi: 10.4155/fsoa-2017-0149 (PMC5905631; doi:10.4155/fsoa-2017-0149)
Supplement: Supplementary file 2 [file fsoa-04-289-s2.doc]

***Appendix – SUPERAR Study Group**

Maria Fabiana Antonelli

Carlos Ascimani Peña

Nora Aste

Carmen Baied

Carlos Baruzzo

Cristian Alejandro Benítez

Ana Maria Beron

César caprarulo

Yung Chi Ju

Adriana Karina Cogo

Silvana Conti

Analia Dellepiane

Julia Demarchi

Maximiliano Fenucci

Óscar Fernández Carro

Rosana Gallo

Rodrigo García Salinas

Julieta Gentiletti

Analia Gervilla Galan

Marisa Jorfen

Carolina Ledesma

Miguel Alex Linarez

Samanta Malm-Green

Alberto Ortiz

Gonzalo Pacheco

Isabel Pineda

Patricia Pinter

Cristina Prigione

Norberto Javier Quagliato

Rosana Quintana

Silvia Rodriguez

Claudia Sa

Leandro Jose Saavedra

Mariana Salcedo

Gabriela Salvatierra

Mariano Cesar Santillan

Juan Jose Scali

María Marcela Schmid

Juan Soldano

Luis Fernando Somma

Enrique Soriano

Edson Velozo

Johana Zacariaz Hereter

Silvia Alejandra Zarate
